# Deferoxamine-Loaded Chitosan-Based Hydrogel on Bone Implants Showing Enhanced Bond Strength and Pro-Angiogenic Effects

**DOI:** 10.3390/jfb15040112

**Published:** 2024-04-22

**Authors:** Huan Liu, Kai Li, Deliang Yi, Yi Ding, Yanfeng Gao, Xuebin Zheng

**Affiliations:** 1 School of Materials Science and Engineering, Shanghai University, 99 Shangda Road, Shanghai 200444, China; liuuhuuan@163.com; 2 Key Laboratory of Inorganic Coating Materials CAS, Shanghai Institute of Ceramics, Chinese Academy of Sciences, 1295 Dingxi Road, Shanghai 200050, China; yideliang@mail.sic.ac.cn (D.Y.); dingyi@mail.sic.ac.cn (Y.D.); 3 Center of Materials Science and Optoelectronics Engineering, University of Chinese Academy of Sciences, 19 Yuquan Road, Beijing 100049, China

**Keywords:** plasma sprayed Ti, chitosan, polydopamine, deferoxamine, angiogenesis

## Abstract

Angiogenesis is vital for bone fracture healing and plays a significant role in the fate of orthopedic implants. The growth and maintenance of new blood vessels at the fracture site of patients is essential, which promotes the clinical outcome of plasma sprayed Ti (PST) coated orthopedic implants. In order to endow the PST coating with pro-angiogenic effects, deferoxamine-loaded chitosan-based hydrogel was fabricated on the coating surface. Polydopamine-modified chitosan (CS/PDA) hydrogel exhibited enhanced bonding strength to PST coatings as evidenced by scratch test. The deferoxamine-loaded CS/PDA (CS/PDA-DFO) exhibited a sustained drug-release property, and the cumulative concentration of released DFO reached 20.21 μg/mL on day 7. PST-CS/PDA with higher wettability and active group quantity enhanced the viability and adhesion characteristics of human umbilical vein endothelial cells (HUVECs) and upregulated the secretion level of nitric oxide and vascular endothelial growth factor. Moreover, the introduction of DFO in PST-CS/PDA further enhanced the pro-angiogenic effects. Above all, this study offers a novel approach for developing hydrogel coating on orthopedic implants showing enhanced bonding strength and pro-angiogenic effects.

## 1. Introduction

Titanium (Ti) and its alloys are commonly employed as orthopedic implants because of their excellent mechanical performance and biocompatibility [1]. In order to enhance the mechanical chimerism between Ti-based implants and bone tissue, plasma-sprayed Ti (PST) coatings are fabricated on implant surfaces. After bone replacement surgery, the local environment loses mechanical stability. New bone and vessels must be generated simultaneously to restore homeostasis and ensure a strong tissue–biomaterial interface, which is essential for permanent implants. However, plasma-sprayed titanium coatings are bioinert and have limited ability to induce active vascular growth [2,3]. Therefore, it is necessary to endow the PST coating with pro-angiogenic effects. 

Deferoxamine (DFO) is a hypoxia-mimetic reagent approved by the US Food and Drug Administration to restore compromised angiogenesis [4]. Specifically, DFO can simulate a hypoxic environment that up-regulates hypoxia-inducible factor-1α (HIF-1α) signal transduction and the expression level of its downstream angiogenic factor vascular endothelial growth factor (VEGF) [5,6,7]. Li et al. [8] loaded DFO via caffeic acid on the Ti surface and demonstrated that DFO upregulated the expression of HIF-1α in MSCs and HUVECs by receptors of TGF-β and TNFR on cell membranes. However, directly loading DFO on an orthopedic Ti implant is challenging in clinical applications as a result of the short half-life of DFO and the initial burst release [9,10]. Therefore, designing a controlled release system for DFO on the PST coating surface is an alternative approach.

Chitosan (CS) is a natural aminopolysaccharide that can experience a reversible sol-gel phase transition at a pH value of 6.3 [11]. In previous studies, CS hydrogel has been widely employed as a drug delivery system since its hydrophilic three-dimensional structure allowed drug load and controlled drug release [12,13,14]. Shi et al. prepared CS-CA hydrogels for the controlled release of taxifolin, exhibiting pH-responsive drug release behaviors [15]. Although the CS hydrogel can be easily obtained at the cathode via electrodeposition [11,16,17], the hydrogel has an inferior bond strength with the underlying substrate [18]. In order to improve the combination between the gel and underlying substrate, an adhesive layer is introduced as a common method. Polydopamine (PDA) has been proven to adhere to nearly any substrate since it shows a structural similarity to the adhesion protein secreted by marine mussels [19,20]. For instance, Liu et al. [21] introduced a PDA coating into carboxymethyl chitosan/sodium alginate-based hydrogel. They observed that the introduction of the PDA coating with catechol groups endowed the hydrogel with improved mechanical properties. Therefore, combining PDA with a CS network has great potential to improve the mechanical performance of CS hydrogel.

In this study, DFO-loaded CS/PDA hydrogel was prepared on PST (PST-CS/PDA-DFO) through cathodic electrodeposition and subsequent DA self-polymerization. The PST and PST-CS/PDA coatings served as controls. The bonding strength between the CS/PDA and the PST was measured through a scratch test. The DFO delivery from the PST-CS/PDA-DFO coating was investigated. The pro-angiogenic effects of the PST-CS/PDA-DFO coating were assessed by measuring the proliferation, adhesion, and angiogenic factors section of HUVECs.

## 2. Materials and Methods

### 2.1. Coating Preparation

**Scheme 1:**
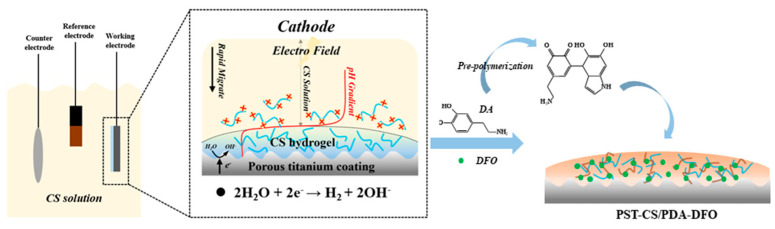
Schematic illustration for the fabrication of the preparation of CS/PDA-DFO hydrogel on the PST coating.

A vacuum plasma spraying system (F4-VB, Sulzer Metco, Pfaeffikon, Switzerland) was employed to prepare porous titanium coatings (PST) on Ti6Al4V. The PST-coated samples were ultrasonically cleaned in alcohol for 10 min and dried at 60 °C. CS hydrogel was obtained on the substrate surface by electrochemical deposition. Specifically, a glacial acetic acid solution was applied to dissolve the CS power to a concentration of 1 wt.% with pH 5.5. Using the three electrodes system for electrode positions, the PST-coated Ti, a piece of platinum, and the calomel electrode were applied as the working electrode, counter electrode, and reference electrode, respectively. Then, PST-coated Ti was placed in 40 mL CS solution, which contained 0.1 wt.% NaCl and 0.1 M H_2_O_2_. A constant current density (10 mA/cm^2^) was used for the electrodeposition of 3 min to obtain PST/CS-coated samples. The PST/CS-coated samples were rinsed with deionized water and immersed in 2 mg/mL dopamine hydrochloride solution, shaken at a speed of 100 rpm for 12 h, and cleaned with deionized water to obtain PST-CS/PDA-coated samples. The as-prepared samples were shaken in DFO solution to obtain DFO-loaded PST-CS/PDA coating. Scheme 1 illustrates the preparation process.

### 2.2. Surface Characterization

The coating surface morphology was characterized by field-emission scanning electron microscopy (FE-SEM, Hitachi, Tokyo, Japan). The elemental composition of the coated samples was detected by X-ray photoelectron spectroscopy (XPS, Escalab 250Xi, Thermo Fisher Scientific, Waltham, MA, USA) with an Al K_α_ source. XPSPEAK41 software was employed to perform peak analysis on C, O, and N elements. An infrared spectrometer (Fourier infrared spectrometer, FTIR, Thermo Scientific, USA) was employed to detect the functional groups of the coatings within 4000–500 cm^−1^.

### 2.3. DFO Release from Hydrogel Coating

The fabricated coatings were soaked in PBS. At pre-determined times (0.5, 1, 2, 4, 16, 24, 48, 72, 120, and 168 h), 2 mL solution was collected and mixed with 2 mL FeCl_3_. A UV spectrophotometer (UV-VIS, UV-4100, Metash, Shanghai, China) was employed to determine the absorbance at 485 nm. The standard curve for DFO was acquired based on the absorbance of different concentrations of DFO. According to the standard curve, the cumulative release efficiency and concentration of DFO were obtained.

### 2.4. Cell Culture

The HUVECs were acquired from the cell bank of the Chinese Academy of Sciences. The DMEM medium containing FBS (10 *v*/*v*%) and penicillin–streptomycin (1 *v*/*v*%) in a cell incubator of 37 °C and 5% CO_2_ was employed to cultivate HUVECs and changed every 3 days. When the HUVECs converged to 80%, the HUVECs at passages three to five were applied for the following experiments.

### 2.5. Cell Morphology Observation

The morphologies of cells were characterized by FE-SEM and confocal laser scanning microscopy (CLSM, TCS SP5 II, Leica, Wetzlar, Germany). HUVECs were incubated on the sterilized coatings at a density of 3 × 10^4^ cells/cm^2^ for 1 day. Glutaraldehyde was used to treat the sample to achieve the goal of immobilizing cells on the surface. The cells were dehydrated using ethanol with a certain gradient and observed by FE-SEM.

For CLSM observation, HUVECs were incubated on the sterilized coatings at 1 × 10^4^ cells/cm^2^ for 1 day. The HUVECs were fixed by paraformaldehyde and dyed with phalloidin and diamidino-2-phenylindole for the cytoskeleton and nucleus, respectively. After PBS cleaning, the cells on the coated samples were observed by CLSM at 405 and 488 nm excitation.

### 2.6. Cell Proliferation Assay

A CCK-8 kit was purchased to evaluate cell proliferation. HUVEC cells were cultured on the sterilized coatings at 1 × 10^4^ cells/cm^2^. After the cultivation of 1, 4, and 7 days, according to the instruction of the CCK-8 kit, a microplate reader (Multiskan Spectrum, Thermo Scientific, USA) was applied to determine the absorbance at 450 nm.

### 2.7. NO and VEGF Secretion

NO kit was purchased to measure the secretion of NO. HUVEC cells were incubated on the sterilized coatings for 3s days at 5 × 10^4^ cells/cm^2^. After collecting the supernatant solution in each well, according to the instructions of the NO kit, the microplate reader was applied to determine the absorbance at 540 nm.

A VEGF kit was purchased to measure the secretion of VEGF. HUVEC cells were incubated on the sterilized coatings for 3 days at 5 × 10^4^ cells/cm^2^. After collecting the supernatant solution in each well, according to the instructions of the NO detection kit, the microplate reader was applied to determine the absorbance at 570 nm.

### 2.8. Statistical Analysis

The mean ± standard deviation (SD) was used by testing three parallel samples. The one-way or two-way ANOVA of GraphPad Prism was applied to ensure the statistical differences in results. The value of *p* was employed to ensure the significance level, namely, *p* < 0.05 as *, *p* < 0.01 as **, and *p* < 0.001 as ***.

## 3. Results and Discussion

### 3.1. Surface Characterization

**Figure 1:**
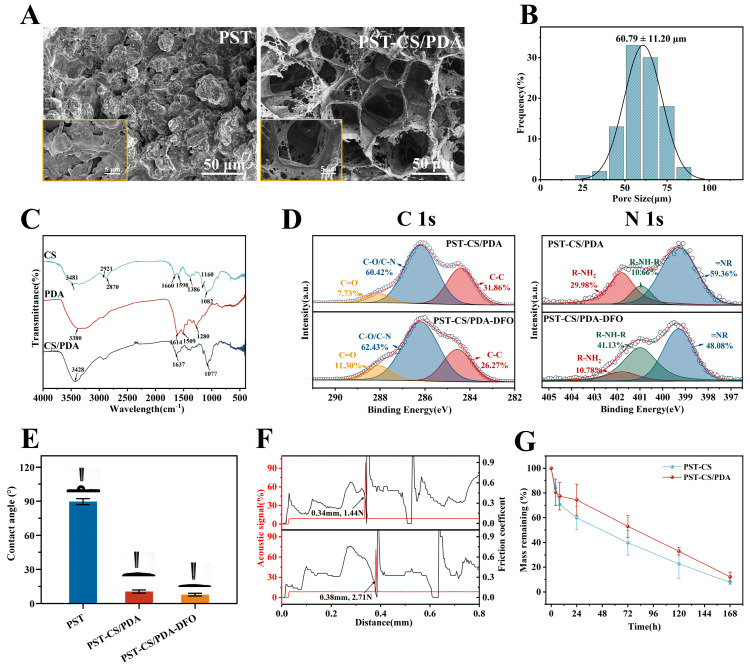
(**A**) The FE-SEM images of PST and PST-CS/PDA. (**B**) Pore size distribution of CS/PDA hydrogel. (**C**) FTIR spectra of CS, PDA, and CS/PDA. (**D**) XPS spectra with peak fitting analysis for C 1s and N 1s of PST-CS/PDA and PST-CS/PDA-DFO. (**E**) Water contact angle of PST, PST-CS/PDA, and PST-CS/PDA coatings. (**F**) The acoustic signal and friction coefficient of PST-CS and PST-CS/PDA. (**G**) Degradation behavior of the PST-CS and PST-CS/PDA.

The surface morphology of the coatings was observed through FE-SEM (Figure 1A). The as-prepared PST coating surface exhibited a rough, porous morphology, which was formed by the accumulation of particles in different melting statuses. The porous surface structure is beneficial for the ingrowth of bone tissue and the formation of mechanical chimerism. PDA-modified CS (CS/PDA) hydrogel was prepared on the PST surface by cathodic electrodeposition and subsequent DA self-polymerization. The CS/PDA hydrogel consisted of interpenetrating macropores with an average diameter of 60.79 ± 11.20 μm, which could be calculated by Image J 1.51j8 (Figure 1B). The macropore structure was beneficial for maintaining the biological activity of the loaded drug. Moreover, the CS/PDA hydrogel was able to prevent the burst release of the drug as a barrier.

Figure 1C displays the FTIR spectra of the CS and CS/PDA hydrogel, which can be used to identify the interaction between CS and PDA. The wide peak appeared at 3481 cm^−1^, corresponding to the stretching vibration of O-H [22] in the CS spectrum. In contrast, the peak at 3481 cm^−1^ shifted to 3428 cm^−1^ in the CS/PDA hydrogel spectrum. The red shift and the increased intensity of the O-H peak indicated the formation of hydron bonds between CS chains [23]. Moreover, the peak at 1082 cm^−1^ shifted to 1077 cm^−1^, and the intensity significantly increased. This phenomenon might be owed to the PDA introduced, which has abundant amine and hydroxyl groups [24]. Furthermore, a peak at 1637 cm^−1^ appeared in the CS/PDA spectrum, which was caused by a Schiff base reaction between the amino group in CS and the quinone in PDA [25]. The spectrum indicated the successful introduction of PDA into CS/PDA hydrogel.

XPS was applied to confirm the introduction of DFO in the CS/PDA hydrogel. The characteristic absorption peaks of C 1s (285.88 eV), N 1s (400.10 eV), and O 1s (532.86 eV) were fitted in the spectra of PST-CS/PDA and PST-CS/PDA-DFO (Figure S1, Supporting Information). The contents of C, N, and O elements are shown in Table S1, Supporting Information. The content of N element increased from 9.03% to 10.31% due to a higher N proportion in the DFO molecule (13.45%) than in the dopamine molecule (9.15%). As shown in Figure 1D, the C1s peaks in PST-CS/PDA and PST-CS/PDA-DFO were divided into three peaks at 284.4, 286.2, and 288.1 eV, corresponding to C–C, C–O/C–N, and C=O, respectively [26,27]. The introduction of DFO increased the content of C=O from 7.73% to 11.30%, which was ascribed to the large amount of C=O in DFO. In Figure 1D, the N1s peak could be divided into three peaks at 401.8, 400.95, and 399.3 eV, corresponding to amine groups (R-NH_2_), lactam groups (R-NH-R) and imine groups (=N-R), respectively [28,29]. The introduction of DFO decreased the content of R-NH_2_ from 29.98% to 10.78% and increased R-NH-R from 10.66% to 41.13%. This result was consistent with the findings in the work by He et al. [30] that the modification of DFO in the chitin composite hydrogel decreased R-NH_2_ content and increased R-NH-R content. These results implied the successful modification of DFO in the CS/PDA hydrogel.

The wettability of the as-prepared coatings was characterized by water contact angle. Figure 1E shows that the water contact angle (CA) value of the PST coating was 89.7 ± 2.55°. As CS/PDA hydrogel was fabricated on the PST coating, the CA values decreased to 10.67 ± 1.37° and 7.9 ± 1.21° for the PST-CS/PDA and PST-CS/PDA-DFO coatings, respectively. The increased hydrophilicity was ascribed to the microporous structure of the hydrogel and a quantity of hydrophilic functional groups in CS and PDA.

As shown in Figure 1F, a scratch test was employed to determine the adhesion between the hydrogel and the PST coating [31,32]. The mesoporous structure of the hydrogel surface resulted in a large fluctuation in the friction coefficient. Based on the results of the sound signal and friction coefficient, it was found that the crack scratch of PST-CS occurred at 340 μm with a critical load of 1.44 N. After the introduction of PDA, the crack position increased to 380 μm, and the loading force increased to 2.71 N. These indicated that the introduced PDA effectively enhanced the bonding strength between CS and PST coating.

The in vitro degradation of hydrogel coating was tested in PBS. Figure 1G shows that both the samples degraded gradually in 7 days. Compared with that of PST-CS, the degradation of PST-CS/PDA was retarded to 87.7%, which can be attributed to the chemical crosslinking of PDA and CS. Meanwhile, a hydrogel with a slower degradation rate is more suitable as a drug-release carrier.

### 3.2. DFO Release from the PST-CS/PDA-DFO Coating

**Figure 2:**
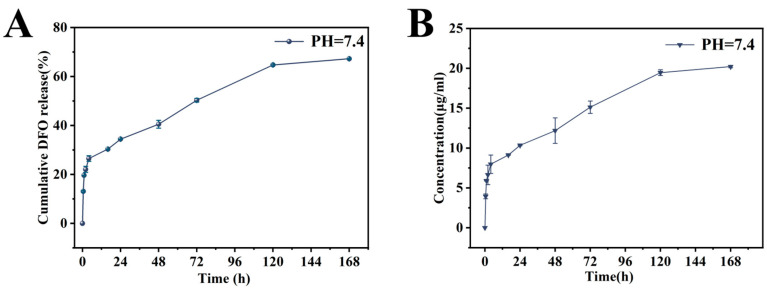
(**A**) Cumulative DFO release of PST-CS/PDA-DFO coating in PBS. (**B**) The cumulative release concentration of DFO.

As shown in Figure 2A, the PST-CS/PDA-DFO coating showed an initial burst release of DFO at 24 h, considering that cumulative drug release reached approximately 34.41%. This was probably because the absorbed DFO, a kind of hydrophilic small molecule, could easily be released through the macropores of the hydrogel at the beginning. The PST-CS/PDA-DFO coating showed a constant release of DFO from day 1 to day 7 and reached 67.24% cumulative drug release on day 7. This phenomenon was caused by the disassembly of the Schiff base crosslinking and subsequent degradation of the hydrogel. The drug release sustained over 8 days, which might fulfill the angiogenesis requirement for orthopedic implants in the clinic.

The cumulative released DFO concentration is shown in Figure 2B. The drug concentration achieved 3.39 μg/mL within 0.5 h and reached equilibrium on the 7th day with a maximal value of 20.21 μg/mL. The released DFO concentration in our study fell within the range of effective angiogenic concentration, as reported previously. In the work by Shao et al., DFO was loaded in gelatin microspheres, which were embedded in PVA-modified chitosan hydrogel. They found that DFO with a concentration of 1.97 μg/mL exhibited great improvement in both HUVEC proliferation and tube formation [33].

### 3.3. Combined Effects of CS/PDA Hydrogel and DFO on the HUVEC Behaviors

**Figure 3:**
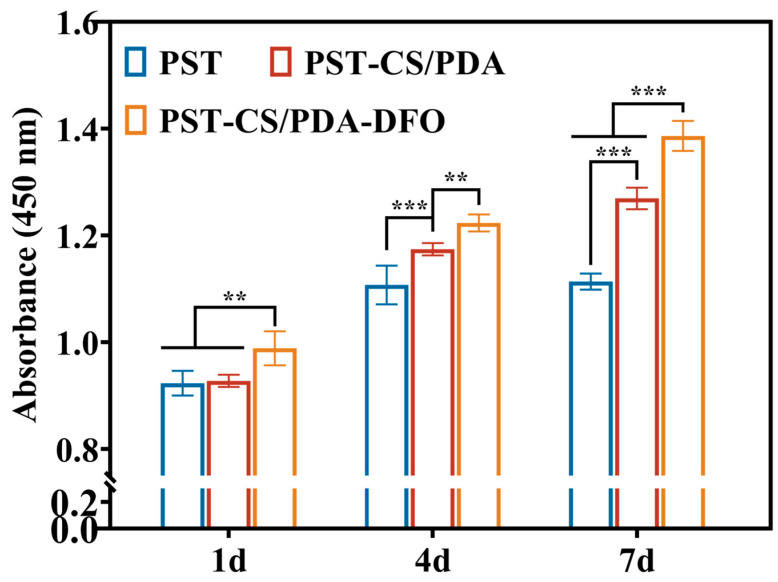
The CCK-8 assay of HUVECs incubated on each group for 1, 4, and 7 days, *n* = 3, ** *p* < 0.01, *** *p* < 0.001.

The proliferation of HUVECs in the coatings was quantified by CCK-8 assay. Figure 3 shows that the OD values of HUVECs cultivated on the surface of all samples increased gradually with time, demonstrating the good cytocompatibility of the tested materials. Compared with the PST coating, the PST-CS/PDA coating significantly improved the proliferation of HUVECs at the predetermined time. Moreover, when DFO was loaded into the hydrogel coating, the proliferation level of HUVECs was further upregulated. PDA and DFO contain a quantity of active groups, such as -NH2 and -OH, which could effectively promote cell growth. In addition, Baccakova et al. suggested that PDA could provide good cell adhesion, which facilitates cell proliferation [34]. Therefore, the highest level of cell proliferation on the PT-CS/PDA-DFO coating was caused by a combination of the CS/PDA multilayer and the released DFO.

**Figure 4:**
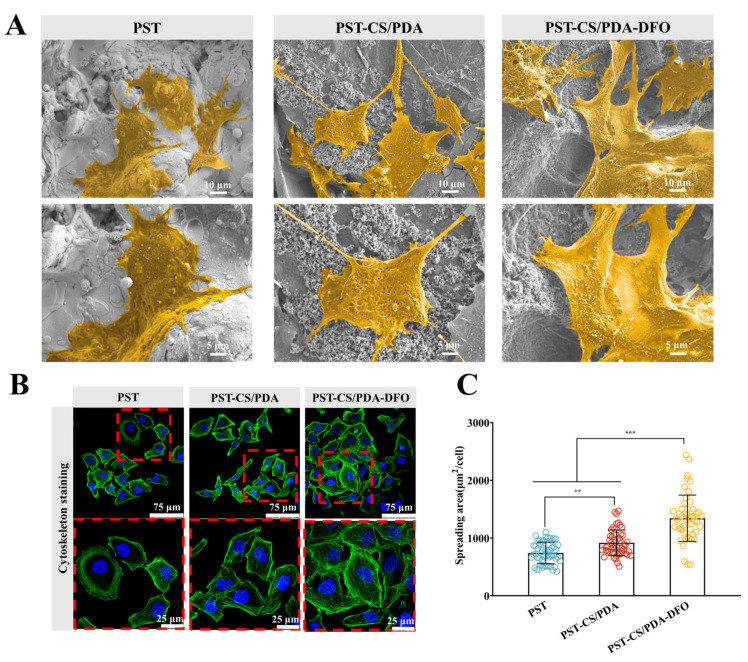
HUVECs cultured on PST, PST-CS/PDA, and PST-CS/PDA-DFO. (**A**) FE-SEM and (**B**) CLSM observation of cell adhesion morphology. (**C**) Quantitative analysis of cell spreading area, *n* = 3, ** *p* < 0.01, *** *p* < 0.001.

Cell adhesion and spreading play an important role in cell migration, proliferation, and differentiation [30]. Thus, the morphology of HUVECs, which were inoculated for 24 h, was observed by FE-SEM and CLSM. Figure 4A displays that the HUVECs on the PST coating surface had limited spread and barely pseudopodia presented. In comparison, the HUVECs cultivated on the PST-CS/PDA exhibited a polygon-shaped morphology and spread more filamentous pseudopodia, which can come into contact with adjacent cells. The introduction of DFO in the PST-CS/PDA coating significantly increased the number of cells. Moreover, the cells stretched with well-developed cell cytoskeletons and clusters, extending more obvious lamellipodia, presenting the greatest connection and the best adhesion morphology.

CLSM images in Figure 4B show the cytoskeleton actin and nucleus exhibited as red and blue, respectively. HUVECs cultured on PST and PST-CS/PDA showed similar polygon shapes. However, the actin fibers and cell microtubules around the nucleus were significantly increased on PST-CS/PDA. Compared with those on the PST and PST-CS/PDA coatings, the cells on PST-CS/PDA-DFO showed more myofilament and clustered branches, as well as a larger cell spreading area. Quantitative analysis results of the cell spreading area are shown in Figure 4C. Compared with the PST coating, the PST-CS/PDA coating slightly increased the spreading area of HUVECs. However, the introduction of DFO increased the cell spreading areas by 1.81- and 1.46-fold for PST and PST-CS/PDA, respectively. These results suggested that the presence of CS/PDA hydrogel facilitated HUVEC adhesion, which might have resulted from increased wettability and the large amounts of -NH_2_ and -OH groups. The increased wettability is beneficial for the absorption culture medium and provides more cell adhesion sites; the chemical groups can stimulate the growth of HUVECs. Introducing DFO into the CS/PDA hydrogel further enhanced HUVEC adhesion. This was probably because of the outstanding biological activity of DFO.

**Figure 5:**
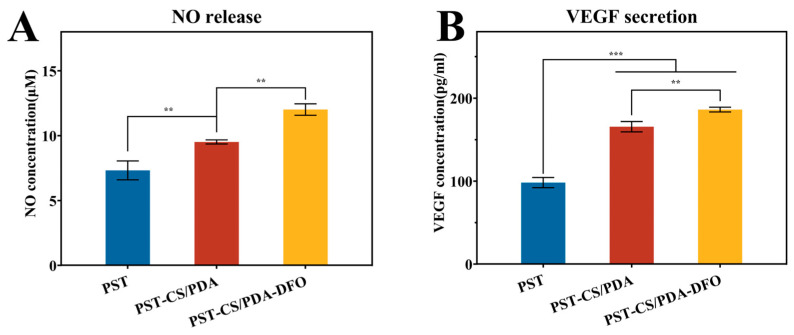
(**A**) NO secretion and (**B**) VEGF secretion of HUVECs cultured on PST, PST-CS/PDA, and PST-CS/PDA-DFO for 3 days, *n* = 3, ** *p* < 0.01, *** *p* < 0.001.

As a vascular endothelial relaxing factor, NO serves as a regulatory signal for angiogenesis [8]. Related studies have shown that the pro-angiogenic effects of NO were mainly through promoting embryonic cells to be involved in new blood vessel formation [4]. Meanwhile, VEGF signaling typically represents a crucial procedure during the physiological process of angiogenesis [35]. The results of NO and VEGF secretion from HUVECs on different coatings are shown in Figure 5. As shown in Figure 5A, PST-CS/PDA significantly increased the NO content of HUVECs compared with PST. Notably, the HUVECs on PST-CS/PDA-DFO possessed the highest NO content, which was 1.26- and 1.64-fold that of the PST and PST-CS/PDA, respectively. The ELISA results shown in Figure 5B revealed that the level of VEGF of the PST-CS/PDA-DFO coating significantly exceeded that of PST and PST-CS/PDA. The secretion of NO and VEGF both implied that the DFO-containing hydrogel coating could better improve the function of HUVECs.

The upregulated pro-angiogenic effects of the PST-CS/PDA-DFO coating were owed to the combined effects of CS/PDA hydrogel and DFO. CS/PDA hydrogel showed enhanced bond strength and prominent biocompatibility. Moreover, the hydrophilic macroporous structure acted as a carrier as well as a diffusion barrier for DFO. The sustained release of DFO in the following 7 days could reach effective concentration and promote NO and VEGF secretion from ECs through the HIF-1 signaling pathway, which is of great importance to HUVECs’ functionalization.

## 4. Conclusions

In this study, we deposited PDA-modified CS hydrogel on the PST coating surface, which served as a DFO carrier. Compared with the CS hydrogel, the CS/PDA hydrogel exhibited enhanced bonding strength to the PST coating due to the large amounts of catechol of PDA. The PST-CS/PDA-DFO coating displayed a sustained drug-release property, and the released DFO concentration within 7 days fell in the range of effective pro-angiogenic concentration as reported previously. The PST-CS/PDA had higher wettability and large amounts of -NH_2_ and -OH bioactive groups, which collaboratively facilitated HUVEC adhesion as characterized by obvious lamellipodia pseudopodia. The PST-CS/PDA-DFO further increased the viability of HUVECs and enhanced their pro-angiogenic effects by up-regulating the secretion of NO and VEGF. Above all, DFO-loaded CS/PDA hydrogel with enhanced bond strength and pro-angiogenic effects shows potential in ameliorating angiogenesis at the implant site.

## Data Availability

Data are contained within the article.

## Supplementary Materials

The following supporting information can be downloaded at: https://www.mdpi.com/article/10.3390/jfb15040112/s1, Figure S1: XPS wide scan C 1s, O 1s, and N 1s spectra of PST-CS/PDA and PST-CS/PDA-DFO; Table S1. The element content of the coatings.
