# Supplementary material for: Deferoxamine-Loaded Chitosan-Based Hydrogel on Bone Implants Showing Enhanced Bond Strength and Pro-Angiogenic Effects
Source: J Funct Biomater. 2024 Apr 22;15(4):112. doi: 10.3390/jfb15040112 (PMC11051205; doi:10.3390/jfb15040112)
Supplement: Supplementary file 1 [file jfb-15-00112-s001.zip › jfb-2929181-supplementary file updated 0423.pdf]

Supplementary Material

Deferoxamine-Loaded Chitosan-Based Hydrogel on Bone Implants Showing Enhanced Bond Strength and Pro-Angiogenic Effects

Huan Liu [1], Kai Li [2,3,*], Deliang Yi [2,3], Yi Ding [2], Yanfeng Gao [1,*], Xuebin Zheng [2,3,*]

[1]School of Materials Science and Engineering, Shanghai University, 99 Shangda Road, Shanghai 200444, China

[2]Key Laboratory of Inorganic Coating Materials CAS, Shanghai Institute of Ceramics, Chinese Academy of Sciences, 1295 Dingxi Road, Shanghai 200050, China

[3]Center of Materials Science and Optoelectronics Engineering, University of Chinese Academy of Sciences, 19 Yuquan Road, Beijing 100049, China

Corresponding authors: likai@mail.sic.ac.cn, yfgao@shu.edu.cn, xbzheng@mail.sic.ac.cn

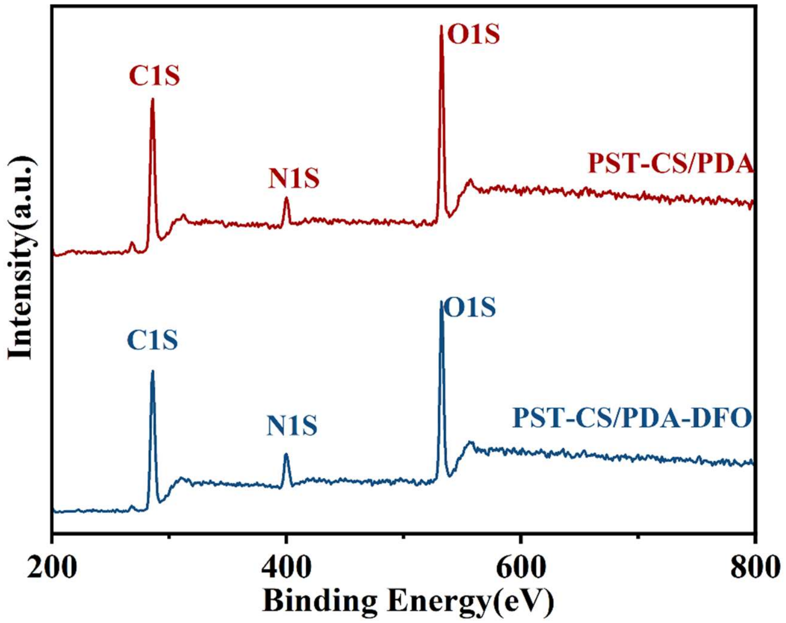

Figure S1. XPS wide scan C 1s, O 1s, and N 1s spectra of PST-CS/PDA and PST-CS/PDA-DFO.

Table S1. The element content of the coatings.

Sample	C (at. %)	O (at. %)	N (at. %)
PST-CS/PDA	61.14	29.84	9.03
PST-CS/PDA-DFO	59.12	30.58	10.31
